# Chemoradioimmunotherapy of inoperable stage III non-small cell lung cancer: immunological rationale and current clinical trials establishing a novel multimodal strategy

**DOI:** 10.1186/s13014-020-01595-3

**Published:** 2020-07-09

**Authors:** Lukas Käsmann, Chukwuka Eze, Julian Taugner, Olarn Roengvoraphoj, Maurice Dantes, Nina-Sophie Schmidt-Hegemann, Sanziana Schiopu, Claus Belka, Farkhad Manapov

**Affiliations:** 1 Department of Radiation Oncology, University Hospital, LMU Munich, Marchioninistrasse 15, 81377 Munich, Germany; 2 Comprehensive Pneumology Center Munich (CPC-M), Member of the German Center for Lung Research (DZL), Munich, Germany; 3 German Cancer Consortium (DKTK), Partner Site Munich, Munich, Germany; 4 Department of Internal Medicine V, Ludwig-Maximilians-University, Munich, Germany

**Keywords:** Non-small cell lung cancer, Chemoradioimmunotherapy, Multimodal treatment, Immunotherapy

## Abstract

Immune-checkpoint inhibitors (ICI) have dramatically changed the landscape of lung cancer treatment. Preclinical studies investigating combination of ICI with radiation show a synergistic improvement of tumor control probability and have resulted in the development of novel therapeutic strategies. For advanced non-small cell lung cancer (NSCLC), targeting immune checkpoint pathways has proven to be less toxic with more durable treatment response than conventional chemotherapy. In inoperable Stage III NSCLC, consolidation immune checkpoint inhibition with the PD-L1 inhibitor durvalumab after completion of concurrent platinum-based chemoradiotherapy resulted in remarkable improvement of progression-free and overall survival. This new tri-modal therapy has become a new treatment standard. Development of predictive biomarkers and improvement of patient selection and monitoring is the next step in order to identify patients most likely to derive maximal benefit from this new multimodal approach. In this review, we discuss the immunological rationale and current trials investigating chemoradioimmunotherapy for inoperable stage III NSCLC.

## Introduction

Stage III non-small cell lung cancer (NSCLC) represents a very heterogeneous disease in terms of patient and tumour characteristics [1–5]. An interdisciplinary approach is necessary to define multimodal treatment strategies based on patients’ condition and disease extension [6]. The majority of these patients are inoperable and multimodal therapy is considered as the cornerstone of treatment [7–10]. Historically, administering platinum-based chemotherapy sequentially or concurrently to thoracic irradiation resulted in a modest improvement of local control, metastasis-free and overall survival compared to radiation alone [11]. As a result of the ground-breaking phase III PACIFIC trial, PD-L1 inhibition with durvalumab after completion of platinum-based concurrent chemoradiotherapy (CRT) which demonstrated historically unprecedented long-term patient outcome is the new standard of care in inoperable stage III NSCLC [8].

Over the last couple of years, immune checkpoint inhibition (ICI) has become an established antitumor treatment in non-driver mutated advanced NSCLC. In 2015, the first PD-1 inhibitor (nivolumab) was approved by the U.S. Food and Drug Administration (FDA) for previously treated advanced or metastatic squamous and non-squamous NSCLC [12, 13]. Subsequently, a combination of pembrolizumab with different chemotherapy regimens (KEYNOTE-189 and 407 trials) demonstrated a further significant improvement of patient survival irrespective of tumour cell PD-L1 status compared to conventional chemotherapy alone [14, 15].

A growing body of evidence from preclinical studies suggest a combination of ICI and radiation as a potential opportunity to achieve a synergistic anti-tumour effect [16]. In this review, we summarise the preclinical data emphasising the rationale on combining chemo-, radio- and immunotherapy, discuss the results of the current studies concerning this trimodal approach in stage III NSCLC, and reveal future perspectives as well as challenges of this novel multimodal treatment strategy.

### Preclinical rationale of combining immune check-point inhibition with chemo- and radiotherapy

For decades, radiotherapy (RT) has been established as an effective local treatment modality. The principal target of ionising radiation is DNA, leading to a potential and fatal cell death [17]. Radiation-induced DNA damage include base damage, single strand breaks, double strand breaks of varying complexity and DNA crosslinks which occur by direct ionisation or indirectly by free oxygen radicals [18]. DNA repair mechanisms could resolve radiation-induced damage in normal cells. However, tumour cells have limited repair capacity and may undergo cell death by radiation-induced cell stress [17]. As a result, regression or complete remission of the irradiated tumour can be observed.

**Fig. 1:**
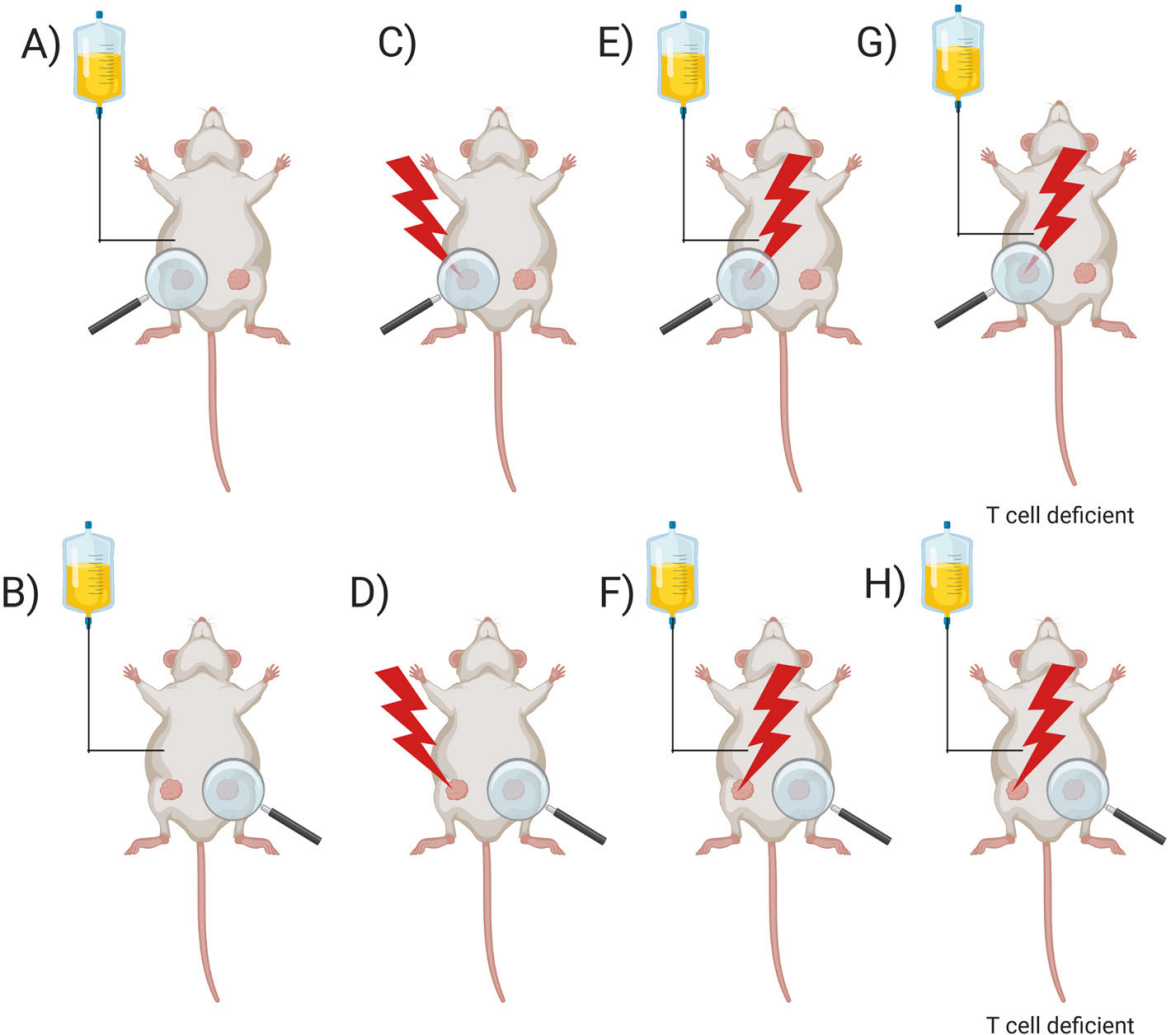
Abscopal effect in preclinical model adapted from Demaria et al. [18]: Mice bearing a syngeneic mammary carcinoma (67NR) in both flanks were treated with growth factor Flt3-Ligand (Flt3-L) or local radiation therapy to one of the two tumors or combined treatment. The Flt3-L was used to enhance the number of available dendritic cells. Administering Flt3-L had no effect on tumor growth delay (**a**, **b**). RT alone led to tumor growth delay of the irradiated tumor (**c**, **d**). Combination treatment resulted in tumor growth delay in both flanks (**e**, **f**) in contrast to T cell deficient mice where no tumor growth delay of nonirradiated tumor was observed (**g**, **h**)

**Fig. 2:**
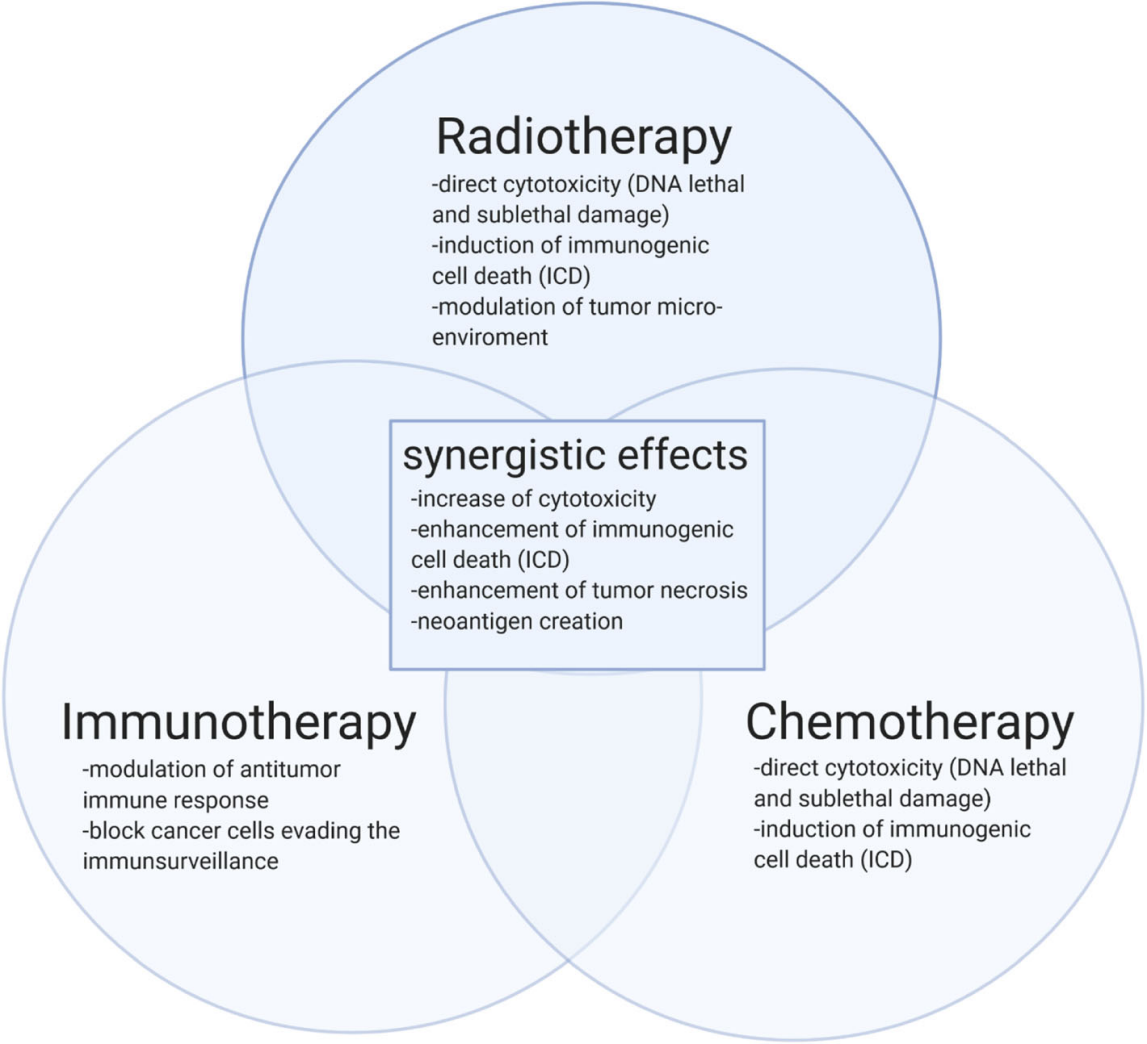
Potential synergistic effects of chemo-, radio- and immunotherapy combinations

**Fig. 3:**
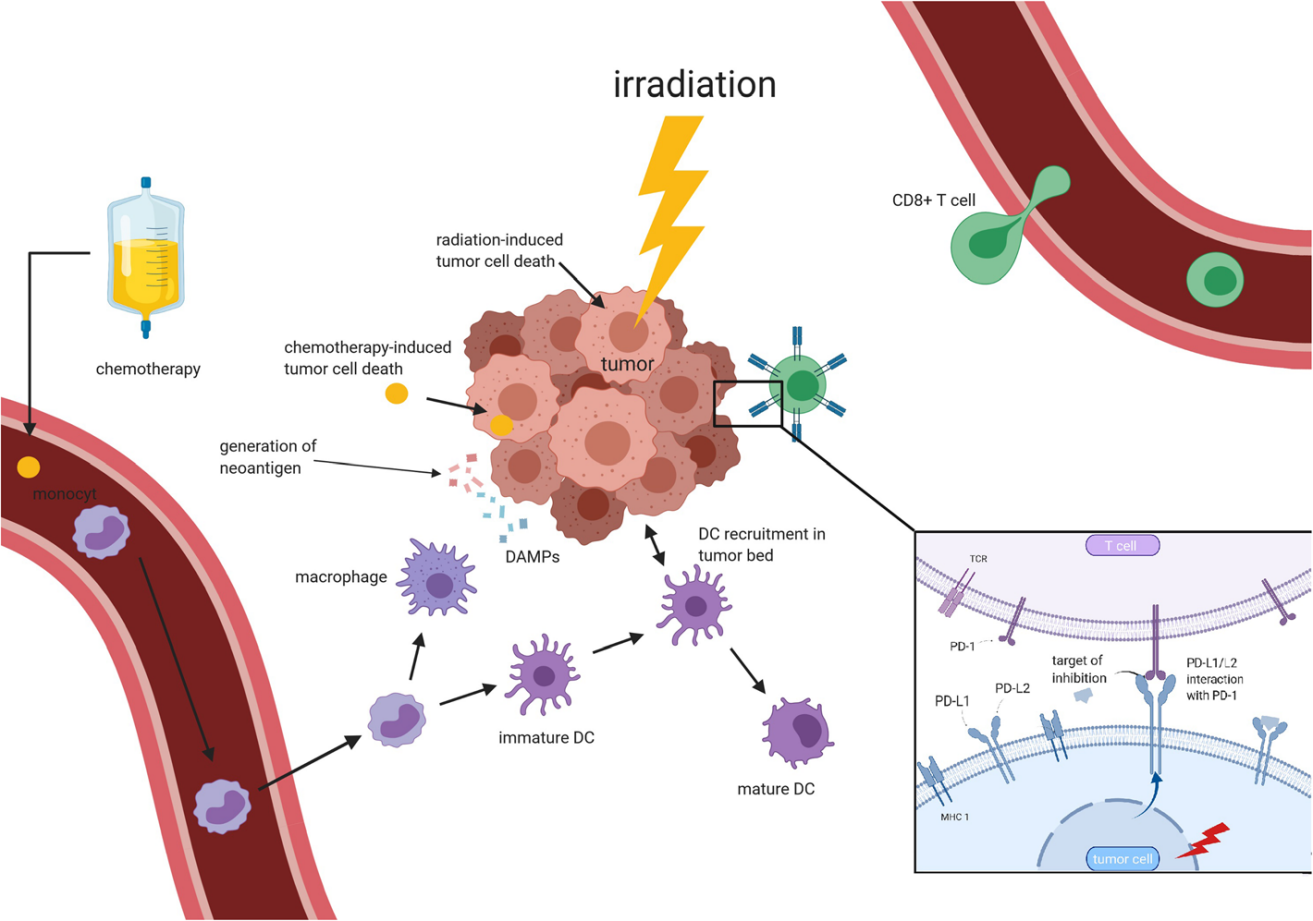
Schematic view of synergistic interactions of chemo-, radio- and immunotherapy at irradiation site adapted from Lauber et al. [17]

Increasing evidence also shows that RT influences tumour lesions outside the irradiated field. This phenomenon was described as the abscopal effect and has been observed for decades, but knowledge and understanding of the underlying mechanisms still remain very limited [19–22]. In 2004, Demaria et al. revealed that the abscopal effect is an immune-modulating effect of RT using a syngeneic mammary cancer model [23] (see Fig. 1). Since then, several preclinical studies have demonstrated that RT induces immunomodulatory changes in the tumour microenvironment (TME), supporting a synergistic potential of radio-immunotherapy [24–29] (see Fig. 2). Combining RT with immune checkpoint inhibition may enhance local and systemic immune-mediated effects and trigger abscopal phenomena. Several mechanisms of radiation-induced immune-modulation have been identified (see Fig. 3) such as a radiation-induced alteration of tumour immunogenicity, generation of pro-inflammatory cytokines and local infiltration of effector cells. Increased major histocompatibility complex (MHC)-I expression after irradiation has been detected in lung cancer, which may lead to enhanced antigen presentation to immune effector cells such as dendritic cells or CD8+ T-lymphocytes [29–31]. Furthermore, irradiation leads to an upregulation of tumour cell PD-L1 expression in lung cancer, which interestingly appears to be CD8+ T-cell–dependent [32–34]. RT also increases natural killer group 2 member D (NKG2D) ligands in NSCLC which aids in tumour cell killing by interacting with NKG2D receptors on NK, NKT and γδ T cells [35, 36]. In addition, RT has been shown to influence T cell priming and activation of antigen-presenting cells such as dendritic cells (DCs) in melanoma mouse model [37]. Thoracic irradiation increases the production of inflammatory cytokines in the lung such as tumour necrosis factor (TNF), interleukin (IL)-1α, and IL-6 in vitro and may be associated with the abscopal effect of tumour response outside the radiation field [38, 39]*.* Furthermore, RT may play an essential part by the generation of tumour-derived antigens including neo-antigens which could be recognised by antigen-presenting cells such as DCs and macrophages. Three immunogenic components are essential for radiation-induced immunogenic cell death (ICD), namely calreticulin, release of high mobility group box 1 (HMGB1) protein and adenosine-5′-triphosphate (ATP) [40]. Importantly, the release of pro-inflammatory cytokines (see Table 1) together with the radiation-induced change of the TME as well as angiogenesis result in the infiltration of activated CD8+ T cells [42, 43] which have been known to facilitate local and distant immune effects of RT [44, 45].

**Table 1:** Potential biomarkers of immunogenic cell death (ICD) adapted from Käsmann et al. [41]

Parameter	Molecular determinants
**Cancer cell-associated pro-tumorigenic cytokines**	IL1, IL10, IL6, IL33, TGF-β, VEGF, VEFGC, IDO enzyme, CXCL12, IL18
**Immune cell-associated pro-tumorigenic cytokines**	IL10, IDO enzyme, TGF-β, IL4, IL5, IL13, TNFα, M-CSF, GM-CSF, IL26, CXCI5, CCL7
**Danger signals (cell surface marker, cytokines/chemokines)**	HMGB1, HSP70, antibodies against calreticulin/HSP90
**Cancer cell-associated viral response-like chemokine signature**	IFN-α, IFN-β, IFN-γ, CXCL9, CXCL10, CXCL1, CCL2
**Immune cell-associated anti-tumorigenic cytokines or chemokines**	IL1B, IL12p70, IL15, IFNG, IL22, IL23, IL17A, IL2, CCL1, CXCL2, CCL4, CCL5, CCL8, CXCL11, CCL12, CCL13, CXCL13, CXCL16, CCL17, CCL19, CCL22, CCL23, CCL24, CCL26

Besides the immunomodulatory effects of RT, chemotherapy has been found to contribute to tumour immunogenicity and tumour immune response [25, 46, 47].

Platinum compounds (cisplatin/carboplatin), etoposide, vinorelbine, pemetrexed and paclitaxel are the most frequently administered systemic agents in NSCLC [1, 3]. Despite their established immunosuppressive effects due to bone marrow suppression, immunoregulatory function which may contribute to antitumoral effects has been postulated [46]. Nowadays, it is hypothesised that conventional chemotherapy can promote tumour immunity due to the modulation of antitumor T cell response through increasing tumour antigenicity, inducing ICD, disrupting immune suppressive pathways and enhancing effector T-cell response.

Platinum compounds are the most studied anticancer agents and still represent the backbone of cancer treatment. Several mechanisms have been identified which show that platinum compounds such as cisplatin could modulate the immune system by the release of tumour antigens and the emission of danger-associated molecular patterns (DAMP) in the TME (see Table 1), upregulation of MHC class I expression, promoting recruitment and proliferation of effector cells, upregulation of cytotoxic effectors and downregulation of the immunosuppressive microenvironment [25, 47, 48]. As a result of the synergistic pathways of platinum compounds with irradiation and radio-sensitising effects, platinum-based chemotherapy appears to be a highly promising candidate of multimodal treatment based on preclinical data [25, 49, 50]. Based on the findings in breast cancer by Golden et al., ICD is produced dose-dependent by irradiation and could be enhanced by the combination of platinum compounds in vitro [40].

Etoposide is a chemotherapeutic agent that inhibits DNA topoisomerase II with resultant DNA strand breaks and induction of cytotoxic and apoptotic cell death [51]. Importantly, etoposide is relatively cell cycle specific, and it affects cells in the S and G2 phases of cell division.

The immunosuppressive property of etoposide is well known due to myelosuppression; however, its immunomodulatory function is not fully understood. Johnson et al. found that etoposide causes apoptosis of activated, but not resting lymphocytes in vitro and indirectly suppresses inflammatory cytokine levels [52]. These findings go along with previous reports [53, 54] which highly question the role of etoposide in combined treatment approaches with immune checkpoint inhibition.

Vinorelbine is a semi-synthetic vinca-alkaloid which represents a spindle poison. The therapeutic mechanism of action is to interfere with the polymerisation of tubulin, a protein responsible for building the microtubule system which appears during cell division. The immunomodulatory properties of vinorelbine are rather unknown. However, vinorelbine in combination with platinum-based chemotherapy has been investigated with potential enhancement of immunogenicity of lung cancer cells. Gameiro et al. showed that MHC class I expression increased more than 50% in H1703 and A549 lung cancer cell lines after treatment with cisplatin/vinorelbine combination [47].

Pemetrexed is an antifolate which inhibits multiple targets involved in folate metabolism. Currently, it is mainly administered in non-squamous NSCLC. In colorectal cancer, pemetrexed treatment alone increased T cell activation in a mouse model, and induced ICD [55]. As a result, pemetrexed is a highly interesting candidate for combination treatment with radiotherapy and immunotherapy due to the increased activity and infiltration of T cells along with the modulation of the innate immune pathways caused by pemetrexed.

Paclitaxel is naturally produced in the bark and needles of *Taxus brevifolia* and represents a tricyclic diterpenoid compound. In contrast to other tubulin-binding chemotherapeutic drugs, paclitaxel promotes the assembly of tubulin into microtubules and prevents the dissociation of microtubules, blocking cell cycle progression, preventing mitosis, and inhibiting tumour growth. Orth et al. found that paclitaxel leads to radiosensitisation via overexpression of mitotic Aurora kinase A (AURKA) and its cofactor Targeting protein for xenopus kinesin-like protein2 (TPX2) [56]. As a result, paclitaxel can increase the rate of apoptosis in tumour cells, release tumour antigens, and may enhance the phagocytosis of antigen-presenting cells. Furthermore, paclitaxel decreases the number and activity of Tregs, increase pro-inflammatory cytokines such as IL-10 and stimulates dendritic cell-mediated antigen presentation [57].

Indeed, growing evidence suggests the immunomodulatory properties of radiotherapy and conventional chemotherapy [23, 40, 58, 59]. Mechanistic evidence such as the induction of ICD by both treatment modalities, the alteration of tumour immunogenicity and the release of pro-inflammatory cytokines by both treatment modalities supports the preclinical rationale that the combination of radiotherapy and chemotherapy enhances immune-mediated antitumor effects (see Fig. 2).

Several immune checkpoint inhibitors have been investigated as monotherapy as well as in combined treatment approaches [58, 60, 61]. At present, the most promising drugs target the cytotoxic T-lymphocyte-associated antigen 4 (CTLA-4) e.g. ipilimumab, tremelimumab and programmed cell death protein 1 (PD-1) e.g. nivolumab, pembrolizumab, sintilimab and its ligand (PD-L1) e.g. atezolizumab and durvalumab. Benefits observed with immunotherapy alone are unfortunately limited to a small population of patients for whom a combination radio- and chemotherapy together with immunotherapy could be beneficial [12, 46, 58, 61–63].

CTLA-4 inhibition has shown considerable antitumor efficacy in melanoma and is still under investigation in NSCLC [58]. CTLA-4 is a member of an immunoglobulin-related receptors family which is expressed by activated T cells and transmits an inhibitory signal to T cells [64]. The complete mechanism of the CTLA-4 pathway remains unclear. However, latest evidence suggests that CTLA-4 recruits a phosphatase to the T cell receptor (TCR) and attenuates the signal [65]. The idea to combine radiotherapy with anti-CTLA-4 antibody (ipilimumab) was supported by a clinical case of a complete and durable abscopal response in metastatic NSCLC [66]. In 2018, Formenti et al. showed that the combination of RT and CTLA-4 inhibition induced systemic anti-tumour T cell response in 21 patients with chemo-refractory metastatic NSCLC [58]. In contrast, anti-CTLA-4 antibodies had failed to demonstrate significant efficacy alone or in combination with chemotherapy.

Based on current preclinical and clinical data, the inhibition of the PD-1/PD-L1 pathway is the most explored immunotherapy strategy in metastatic NSCLC [8, 60, 61, 67–69]. PD-1 regulates T-cell and can limit the activity of antigen-specific T cells in the tumour and TME. PD-1 interacts with two ligands, PD-L1 and PD-L2. If PD-L1, expressed by tumour cells, links with PD-1, expressed by tumour-infiltrating CD8+ T cells, cytotoxic T cell activity will be inhibited, which allows tumour cells to evade immune attack. Immune checkpoint inhibition with anti-PD-1 and anti-PD-L1 antibodies blocks the interaction between PD-L1 and PD-1, leading to enhanced antitumor CD8+ T cell responses. The expression of PD-L1 on tumour cells has been reported to be positively correlated with the efficacy of anti-PD-1/PD-L1 therapy [70]. However, PD-L1-negative tumours were found to respond to PD-L1 inhibition as well [14, 61], questioning the role of PD-L1 expression on tumour cells as a prognostic biomarker alone. The role of host immune cells and PD-L1 expression remains unknown and the demand for more robust biological and imaging predictive biomarkers is high. Interestingly, Reynders et al. found a lower PD-L1 gene expression in tumour samples compared to surrounding non-malignant lung tissue via RNA-sequencing [71]. PD-1 and PD-L1 expression could also be found in peripheral blood mononuclear cells [72, 73]. Importantly, PD-L1 expression of the tumour as well as peripheral blood compounds could vary significantly during and dramatically change after treatment [34, 74–78]. Therefore, we see the need to monitor immune response of immunotherapy and combination treatments. Correlative studies of ICD need to be considered in order to predict tumour response as well as durable tumour control (see Table 1). Fujimoto et al. found that alteration of tumour cell PD-L1 expression after concurrent CRT in locally-advanced NSCLC was significantly associated with patient prognosis [34]. Wang et al. measured PD-L1 expression in circulating tumour cells (CTCs) before radio- or chemoradiotherapy in NSCLC and found PD-L1 positive patients (≥5% of CTCs positive for PD-L1) associated with shorter PFS [79] Patients with an increased PD-L1 expression on tumour cells after CRT had significantly worse overall survival [80]. Dovedi et al. also demonstrated that fractionated RT can lead to increased PD-L1 expression on tumour cells and limit anti-tumor immune response in murine models [32]. As a result, a combination of RT with PD-1/PD-L1 inhibition could be favourable due to restoration of systemic immunity and potentially increased efficacy of immune checkpoint inhibition depending on PD-L1 expression. Combinations of RT and PD-L1 inhibition have been investigated in vivo in several cancer types and resulted in a significantly delayed tumour growth [32, 69].

The Combination of radiotherapy and immunotherapy such as PD-1/PD-L1 inhibition as well as the combination of chemotherapy and immunotherapy have been investigated with synergistic antitumor effects [23, 46, 49, 58, 59, 63, 66, 76, 81–84] (see Fig. 2). Based on the previously described mechanisms of ICD, the combination of all three treatment modalities is supported by the mechanistic evidence. However, preclinical studies administering radiotherapy, chemotherapy and immunotherapy are limited. Recently, Luo et al. reported in three different two-tumour mouse models that concurrent triple therapy with RT, anti-PD-1 and cisplatin resulted in significant enhancement of the abscopal effect via the CXCR3/CXCL10 axis as well as cisplatin-induced CD8+ T cells and cytoreductive effect [63].

In summary, mechanistic evidence clearly suggests a combination of RT with PD-1/PD-L1 inhibition and chemotherapy as a very promising strategy in lung cancer. The synergistic effects of combining radio- and chemotherapy with immunotherapy need to be further investigated. Correlative studies on immune response monitoring need to be implemented.

### Clinical studies reporting on combining immune check-point inhibition with chemoradiotherapy

Several clinical trials are available assessing the combination of PD-1/PD-L1 checkpoint inhibitors with chemoradiotherapy in NSCLC either in the concurrent or sequential setting. www.clinicaltrials.gov was last queried on 01/05/2020 for all trials containing the words “NSCLC OR Non-Small Cell Lung Cancer” in the condition section and “chemoradiotherapy OR radiotherapy OR radiation” in the other terms section.

One thousand one hundred sixty-one trials were identified. After filtering for interventional (clinical trial) studies, 1041 trials remained. Further stratification for trials starting from 01/01/2010 and active as of 01/05/2020 i.e. recruiting OR active, not recruiting OR completed, 382 trials were identified. Twenty studies were selected manually based on relevance (Table 2; note the two parts of the DETERRED study). In addition, PubMed database, Google Scholar and generic internet search was performed for the above-mentioned studies to access abstracts/full publications when available.

**Table 2:** Studies of IO in combination with CRT in inoperable stage III NSCLC

Trial	NCT	Study phase	Number of patients	Status	Trial design	RT details	Median FU (mos)	Median PFS (mos)	Median OS (mos)
**PACIFIC** [85]	NCT02125461	IIIR	713/709 received consolidation	Active, not recruiting	cCRT→durvalumab vs. placebo	54–66 Gy	25.2	17.2	NR
**PACIFIC-2** [86]	NCT03519971	IIIR	300	Active, not recruiting	cCRT + durvalumab→durvalumab vs. cCRT + placebo→placebo	60 Gy	X	X	X
**PACIFIC-5** [87]	NCT03706690	IIIR	360	Recruiting	cCRT→durvalumab fixed dose vs. cCRT→placebo	54–66 Gy	X	X	X
**PACIFIC-6**	NCT03693300	II	150	Recruiting	sCRT→durvalumab	54–66 Gy	X	X	X
**COAST** [88]	NCT03822351	IIR	300	Recruiting	cCRT→durvalumab vs. cCRT→durvalumab + Oleclumab vs. cCRT→durvalumab + monalizumab	54–66 Gy	X	X	X
**DETERRED-PART I** [89, 90]	NCT02525757	II	10	Active, not recruiting	cCRT→CT + atezolizumab→atezolizumab	60–66 Gy	22.5	18.6	22.8
**DETERRED-PART II** [89, 90]	NCT02525757	II	30	Active, not recruiting	cCRT + atezolizumab→CT + atezolizumab→atezolizumab	60–66 Gy	15.1	13.2	NR
**NICOLAS** [91]	NCT02434081	II	82/94 per protocol v2.0/v3.0	Active, not recruiting	3x CT → RT + nivolumab→nivolumab (sCRT arm of v2.0) OR 1x CT → cCRT + nivolumab→nivolumab	66 Gy	13.4	X	X
**HCRN LUN 14–179** [92, 93]	NCT02343952	II	92	Active, not recruiting	cCRT→pembrolizumab	59–66.6 Gy	16.4	15.4	NR
**Rutgers** [94]	NCT02621398	I	21/23 evaluable	Active, not recruiting	cCRT→pembrolizumab (cohort 1: 4 pts) & cCRT + pembrolizumab→pembrolizumab (cohorts 2–6: 19 pts)	60 Gy	16	18.7	29.4
**CLOVER NSCLC**	NCT03509012	I	300 solid tumors (NSCLC, HNSCC, SCLC)	Recruiting	cCRT + durvalumab	X	X	X	X
**KEYNOTE-799**	NCT03631784	II	216	Recruiting	1x CT + pembrolizumab→cCRT + pembrolizumab→pembrolizumab	60 Gy	X	X	X
**H. Lee Moffitt Cancer Center**	NCT03663166	I/II	50	Recruiting	cCRT +2x ipilimumab→nivolumab	60 Gy	X	X	X
**Alliance Foundation**	NCT03102242	II	64	Active, not recruiting	2x OR 4x atezolizumab→cCRT→2x CT → atezolizumab	60 Gy	X	X	X
**EMD Serono**	NCT03840902	IIR	350	Recruiting	cCRT + M7824 → M7824 vs. cCRT + placebo→durvalumab	60 Gy	X	X	X
**CheckMate73L**	NCT04026412	IIIR	1400	Recruiting	cCRT + nivolumab →nivolumab + ipilimumab OR cCRT + nivolumab→nivolumab vs. cCRT→durvalumab	X	X	X	X
**CONSIST**	NCT03884192	IIIR	162	Recruiting	cCRT→sintilimab (IBI308) vs. cCRT alone	X	X	X	X
**CStone Pharmaceuticals**	NCT03728556	IIIR	702	Recruiting	sCRT/cCRT→CS1001 mAb vs. placebo	X	X	X	X
**Sun Yat-sen University**	NCT04085250	IIR	264	Recruiting	CT + nivolumab→cCRT→nivolumab vs. observation	X	X	X	X
**NCI study**	NCT04092283	IIIR	660	Recruiting	cCRT + durvalumab→durvalumab vs. cCRT→durvalumab	X	X	X	X
**BTCRC-LUN16–081**	NCT03285321	IIR	108	Recruiting	cCRT→nivolumab vs. nivolumab + ipilimumab	X	X	X	X

Legend: c - concurrent; CT - chemotherapy; s - sequential; CRT - chemoradiotherapy; R - randomised; Gy - Gray; PFS - progression-free survival; OS - overall survival; NR - not reached; X - not available; FU - follow-up; HNSCC - squamous cell carcinoma of the head and neck (HNSCC); mAb - monoclonal antibody; NSCLC - Non-small cell lung cancer; SCLC - Small cell lung cancer

### Studies on chemoradiotherapy and sequential immune check-point inhibition

#### PACIFIC trial (NCT02125461) [85]

The PACIFIC trial randomly assigned patients after concurrent CRT 2:1 to durvalumab, an anti-PD-L1 human IgG1 monoclonal antibody 10 mg/kg IV or placebo every 2 weeks for up to 12 months. Durvalumab was administered 1 to 42 days after completion of primary multimodal treatment. The co-primary outcome measures were PFS and OS. Secondary endpoints included 12- and 18-month PFS rates, overall response rate (ORR) and safety. Of 713 randomised patients, 709 received consolidation therapy (473 in the durvalumab arm and 236 in the placebo arm). On interim analysis, median PFS from randomisation was 16.8 months with durvalumab vs 5.6 months with placebo; 12-month PFS rate was 55.9% vs 35.3%, and 18-month PFS rate was 44.2% vs 27.0%. Importantly, grades 3–4 toxicity was similar in both groups: 29.9% vs 26.1%. Treatment was discontinued due to adverse events (AEs) in 15.4% of patients in the durvalumab group vs 9.8% in the placebo group.

In an updated analysis published in December 2018, the 24-month OS rate was 66.3% in the durvalumab group vs 55.6% in the placebo group. The updated PFS rates were in accordance with the previously published data. A total of 30.5 and 26.1% patients in the durvalumab and placebo group had grade 3/4 AEs; 15.4 and 9.8% of the patients, respectively, discontinued the trial regimen because of AEs.

Based on the results of the interim analysis, the FDA approved durvalumab on February 16, 2018, for the treatment of patients with stage III NSCLC whose tumours are unresectable and without disease progression following CRT. The European Medicines Agency (EMA) followed suit on September 21, 2018 however choosing to approve the drug for patients with PD-L1 expressing tumours (PD-L1 at least 1% on tumour cells assessed on archived pre-CRT tumour tissue using the VENTANA PD-L1 [SP263] immunohistochemistry assay) based on the results of an unplanned exploratory post-hoc analysis in a small patient subset, that failed to demonstrate an OS benefit in PD-L1 negative tumours. Indeed, a panel of international lung cancer experts have voiced their concern regarding this conditional approval [95].

#### PACIFIC 5 trial (NCT03706690) [87]

The PACIFIC 5 trial is a double-blind, placebo-controlled, multicentre study currently assessing safety and efficacy of fixed-dose durvalumab 1500 mg IV every 4 weeks (in contrast, dosing in the PACIFIC trial was 10 mg/kg IV every 2 weeks) in participants with unresectable stage III NSCLC who have not progressed following definitive, platinum-based concurrent CRT. EGFR or ALK genomic abberations will be capped at approximately 15%. The primary endpoint is PFS. Approximately 360 patients will be randomized 2:1 to receive durvalumab or placebo. Participants will have stable disease (SD) or better following primary multimodal treatment. The study completion date is set for 01/2025.

#### PACIFIC 6 trial (NCT03693300)

The PACIFIC 6 trial is an open-label, multicentre phase II trial currently assessing safety of fixed-dose durvalumab 1500 mg IV every 4 weeks in participants with unresectable stage III NSCLC who have not progressed following definitive, platinum-based sequential CRT (in contrast PACIFIC 5 will assess concurrent CRT). Approximately, 150 patients will be treated with the durvalumab in Europe and North America. Radiation therapy must be completed within 28 days prior to first durvalumab administration. Participants will be treated with durvalumab in two cohorts: approximately 120 participants in the Eastern Cooperative Oncology Group Performance Status (ECOG PS) 0 to 1 cohort and approximately 30 participants in the ECOG PS 2 cohort. The primary outcome measure is the number of participants with Grade 3 and Grade 4 treatment-related AEs. Secondary endpoints include PFS, OS. The estimated study completion date is 02/2023.

#### Hoosier Cancer Research Network (HCRN) LUN 14–179 trial (NCT02343952) [92, 93]

The LUN 14–179 is an open-label, multi-institutional phase II trial of consolidation pembrolizumab following cCRT in patients with unresectable stage III NSCLC. Following platinum-based CRT to a dose of 59–66.6 Gy, patients without disease progression after 4–8 weeks received pembrolizumab 200 mg IV every 3 weeks for up to 1 year. The primary endpoint was median time to metastatic disease or death (TMDD). Secondary endpoints included PFS, OS, and toxicity. Ninety-three patients were enrolled and thus eligible for efficacy analysis. After a median follow-up of 16.4 months, median TMDD was not reached but the estimates of 1-yr and 2-yr OS were 80.5 and 68.7% respectively; median PFS was 15.4 months. 12-, 18-, and 24-month PFS were 59.9, 49.5, and 45.4% respectively. Five (5.4%) had grade 3–4 pneumonitis. There was one pneumonitis-related death. Other than dyspnoea (5.4%), no other grade 3/4 toxicities exceeded 5%. Median number of cycles of pembrolizumab was 13.5 (1–19). Sixteen percent received < 4 cycles; 84% received ≥4 cycles; 37% completed one-year pembrolizumab.

In a subset analysis by Anouti et al., the authors conducted a univariate analysis to evaluate which variables (*p* < 0.1) might be associated with TMDD, PFS and OS and found stage IIIA and ≥ 4 cycles of pembrolizumab; stage IIIA, ≥ 4 cycles of pembrolizumab, and V20 < 20%; stage IIIA and ≥ 4 cycles of pembrolizumab might be associated with improved outcomes for TMDD, PFS, and OS, respectively.

#### DETERRED trial – part I (NCT02525757) [89, 90]

The DETERRED trial is a single institution phase II trial assessing the safety and feasibility of concurrent CRT followed by consolidation full dose carboplatin/paclitaxel (CP) with atezolizumab and maintenance atezolizumab up to 1 year for locally advanced NSCLC. The study consists of 2 parts: part I assessed sequential while part II assessed simultaneous PD-L1 blockade (see simultaneous ICI section of this review). In part I, sequential atezolizumab and CP after completing primary multimodal treatment was assessed in 10 patients. Any grade ≥ 3 AEs was seen in 6/10 patients (60%), with most common being pneumonia (2/10; 20%). Three grade ≥ 3 AEs (30%) were attributed to atezolizumab, including dyspnoea, arthralgia and a grade 5 tracheo-oesophageal fistula. Grade 2 radiation pneumonitis (RP) was seen in 3 patients. Four patients had disease progression during atezolizumab maintenance and had died between 0.93 to 1.86 years. Based on the latest abstract, 4 patients completed atezolizumab and were in follow-up without recurrence.

#### Coast (NCT03822351) [88]

COAST is a phase II, open-label, multi-center, randomised multidrug platform study assessing the efficacy and safety of durvalumab alone vs. durvalumab in combination with novel agents oleclumab or monalizumab in locally advanced, unresectable, stage III NSCLC. Approximately 300 patients will be randomized 1:1:1. Participants will have stable disease (SD) or better following primary cCRT. Estimated completion date is planned in 2023.

#### Alliance foundation study (NCT03102242)

Single arm open-label multicentre non-randomised phase II trial assessing induction immunotherapy with atezolizumab. The study is currently active but not recruiting with an estimated enrolment of 64 subjects. Participants received either 2 or 4 cycles of induction atezolizumab 1200 mg IV every 3 weeks with restaging performed after cycle 2 and cycle 4. In case of progressive disease after cycle 2 and still stage III and eligible for curative-intent therapy, patients were referred for taxane-platinum combination CRT to a dose of 60 Gy otherwise after the 4 planned cycles. After cCRT, 2 cycles of consolidation chemotherapy with carboplatin AUC6 and paclitaxel 200 mg/m^2^ IV every 3 weeks beginning 3–5 weeks after completion of radiation was delivered followed by adjuvant atezolizumab 1200 mg IV every 3 weeks to complete 1 year of therapy from the start of induction. Importantly, anti-PDL1 therapy was interrupted during CRT. The primary endpoint is disease control rate after 12 weeks induction.

#### CONSIST study (NCT03884192)

The CONSIST study evaluates the safety/efficacy of consolidation therapy with sintilimab (IBI308), an anti-PD1 recombinant human monoclonal antibody after cCRT for unresectable, locally advanced stage III NSCLC. In this open-label, multicentre, randomised phase III study, patients receive cCRT followed by observation vs. consolidation sintilimab 200 mg IV every 3 weeks for a maximum of 12 months. The primary endpoint is PFS. Secondary endpoints include OS, ORR, AEs. Approximately 162 patients will be enrolled with an estimated study completion date set for 12/2021.

#### CStone pharmaceuticals (NCT03728556)

This study evaluates the safety and efficacy of consolidation therapy with CS1001, an anti-PD-L1 fully human monoclonal antibody after cCRT for unresectable/locally advanced stage III NSCLC. In this randomised, double-blind, multicentre, placebo-controlled phase III study, participants receive sCRT/cCRT followed by placebo vs. CS1001 1200 mg IV every 3 weeks for up to 24 months. The primary outcome measure is PFS. Approximately 402 participants will be enrolled with an estimated study completion date set for 08/2023.

#### Sun Yat-sen University (NCT04085250)

This study explores the safety and efficacy of nivolumab as consolidation therapy in patients with locally advanced, unresectable stage III NSCLC who have not progressed following neo-adjuvant chemotherapy plus nivolumab and definitive cCRT. In this randomised, open-label phase II study, participants receive 2 cycles of neo-adjuvant therapy comprising docetaxel 75 mg/m^2^ + cisplatin 75 mg/m^2^ + nivolumab 360 mg IV, once every 3 weeks followed by cCRT with docetaxel 25 mg/m^2^ + cisplatin 25 mg/m^2^ IV, once a week for a total of 4 weeks and consolidation nivolumab 480 mg IV every 4 weeks for a maximum duration of 12 months OR observation. The primary outcome measure is PFS. Secondary outcome measures are OS, ORR, AEs, symptoms and health-related quality of life. Approximately 264 participants will be enrolled with an estimated study completion date set for 11/2023.

#### Big Ten Cancer Research Consortium (BTCRC)-LUN16-081 (NCT03285321)

This open-label, multi-centre, randomized phase II investigates consolidation immunotherapy with either nivolumab alone or the combination of nivolumab and ipilimumab following cCRT in inoperable stage III NSCLC. Participants will be randomized to either nivolumab 480 mg IV every 4 weeks for up to 6 cycles or nivolumab 3mg/kg IV every 2 weeks plus ipilimumab 1 mg/kg IV every 6 weeks for up to 4 cycles (in total 12 cycles of nivolumab and 4 cycles of ipilimumab). The primary endpoint is PFS. The secondary endpoints are OS, time to metastatic disease and AEs. An accrual of 108 patients is planned with an estimated study completion date set for 09/2022.

### Studies on chemoradiotherapy and simultaneous immune check-point inhibition

#### Rutgers (NCT02621398) [94]

This multicentre, non-randomised phase I trial using a 3 plus 3 design assessed the safety of timing and dosing of pembrolizumab sequentially and concurrently with taxane-platinum combination definitive photon/proton-based CRT to a dose of 60 Gy. Dose cohorts consisted of full-dose pembrolizumab (200 mg IV every 3 weeks) 2 to 6 weeks after CRT (cohort 1: 4 patients); reduced-dose pembrolizumab (100 mg IV every 3 weeks) starting d29 of CRT (cohort 2: 4 patients); full-dose pembrolizumab starting d29 of CRT (cohort 3: 3 patients); reduced-dose pembrolizumab starting d1 of CRT (cohort 4: 3 patients); full-dose pembrolizumab starting d1 of CRT (cohort 5: 3 patients); and a safety expansion cohort of cohort 5 (cohort 6: 6 patients). Pembrolizumab was then continued every 3 weeks for a year. From 2016 to 2018, 23 patients were enrolled, of whom 21 received at least 1 dose of pembrolizumab and were thus evaluable. The primary endpoint was safety and tolerability. Secondary endpoints included locoregional recurrence, distant-metastasis-free survival, PFS, OS, and rate of pneumonitis. With a median follow-up of 16 months, a median of 7 (range: 0–17) cycles of pembrolizumab were administered. No dose-limiting toxicity was observed, however, 1 G5 pneumonitis occurred in the safety expansion cohort. Grade ≥ 3 immune-related adverse events occurred in 4 patients (18%). Median PFS was 18.7 months, and 6- and 12-month PFS were 81 and 69.7%, respectively.

#### PACIFIC 2 trial (NCT03519971) [86]

The PACIFIC 2 study is a double-blind, multicentre, international randomised study assessing whether durvalumab administered simultaneous to cCRT provides additional benefit, in terms of PFS and ORR vs. cCRT alone. Approximately 300 patients with unresectable stage III NSCLC will be randomised 2:1 analogue PACIFIC to receive either fixed dose durvalumab (1500 mg) every 4 weeks and cCRT vs. placebo and cCRT. Primary endpoints are PFS and ORR (as per RECIST v1.1) assessed via blinded independent central review. Secondary endpoints include OS; OS at month 24; complete response (CR) rate; duration of response; disease control rate; TMDD; time from randomisation to second progression; safety; and symptoms, functioning and global health status. Patients with at least SD will continue to receive durvalumab or placebo until disease progression, or until another discontinuation criterion is met. Study enrolment began in March 2018 and recruitment is ongoing.

#### NICOLAS trial (NCT02434081) [91]

NICOLAS is an open-label, multicentre phase II trial conducted by the European Thoracic Oncology Platform (ETOP) evaluating the safety of platinum-based CRT to a dose of 66 Gy (33 fractions in concurrent and 24 fractions in sequential arm) with concurrent nivolumab in inoperable stage III NSCLC. The protocol initially allowed cCRT or sCRT followed by nivolumab consolidation. Following PACIFIC, demonstrating safety and feasibility of sequential ICI, the trial protocol was amended to address the question of concurrent ICI (v2.0 and v3.0). However, protocol v3.0 only allowed for cCRT. Nivolumab was administered at 360 mg IV every 3 weeks for the first 4 doses (8 doses in sCRT arm of v2.0), followed by 480 mg IV every 4 weeks for up to 1 year.

The primary endpoint was safety defined by 6-month post-radiotherapy pneumonitis rate of grade ≥ 3. A formal interim safety analysis was scheduled when the first 21 patients enrolled reached 3-months follow-up post-radiotherapy. From 08/16 to 08/18, 82 patients per protocol v2.0 and v3.0 were recruited with follow-up up to 13/12/18. Two patients died prior to treatment; thus 80/82 patients were evaluable. For the first 21 patients, no grade ≥ 3 pneumonitis was observed by the end of the 3-month post-radiotherapy follow-up period confirming safety. With a median follow-up of 13.4 months, among the 80 evaluable patients, 8 grade 3 pneumonitis events were observed, with no higher-grade event. Fatal events were observed in 7 patients, of which 1 (autoimmune disorder) was potentially associated with nivolumab. Furthermore, 1-year PFS is planned in the expanded patient cohort of all enrolled patients and is eagerly awaited.

#### DETERRED trial – part II (NCT02525757) [89, 90]

The DETERRED – part II trial is a single institution phase II trial assessing the safety and feasibility of simultaneous PD-L1 blockade with atezolizumab and concurrent CRT followed by consolidation full dose carboplatin/paclitaxel (CP) with atezolizumab and maintenance atezolizumab up to 1 year for locally advanced NSCLC. Thirty patients with inoperable stage III NSCLC were included. Atezolizumab was administered at 1200 mg IV every 3 weeks for up to 1 year from the first dose. Radiation dose at 60–66 Gy in 30–33 fractions was combined with weekly low dose CP, followed by 2 cycles of full dose CP. Severe AEs grade ≥ 3 were defined within 15 weeks of start of primary multimodal treatment or any immune-related AEs during ICB treatment. Evaluable patients had at least one dose of atezolizumab. 17/30 patients had any grade ≥ 3 AEs (57%), with pneumonia being the most common (6/30; 20%). Three (10%) were attributed to atezolizumab (dyspnoea, fatigue and heart failure). Radiation pneumonitis was observed in 3 patients, with 2 grade 2 and 1 grade 3, hence atezolizumab was discontinued. Four patients had progressed and 4 had died, 2 due to disease and 2 due to treatment (neutropenic sepsis and gastric haemorrhage). All other patients had completed primary treatment and were on maintenance atezolizumab (5–19 doses). Updated efficacy results are pending however, this result suggests feasibility of concurrent atezolizumab administration with concurrent CRT followed by maintenance treatment with atezolizumab.

#### Clover (NCT03509012)

The CLOVER trial is an open-label, multicentre phase I trial currently assessing safety and tolerability of durvalumab +/− tremelimumab in combination with cCRT in patients with squamous cell carcinoma of the head and neck (HNSCC), NSCLC and SCLC. In the NSCLC arms, patients with unresectable stage III NSCLC receive a platinum doublet with durvalumab concurrently. Approximately 360 participants are planned and will be enrolled in North America, Europe and Asia. Primary endpoints are number of subjects with dose limiting toxicities (DLTs) and adverse events.

#### KEYNOTE-799 (NCT03631784)

The KEYNOTE-799 trial is an open-label multicentre non-randomised phase II trial of pembrolizumab in combination with a platinum doublet chemotherapy regimen. Patients will receive 1 cycle of pembrolizumab 200 mg IV on d1 with carboplatin/paclitaxel or cisplatin/pemetrexed and approximately 3 weeks later cCRT with the same regimens to a dose of 60 Gy with 2x concurrent pembrolizumab followed by 14 additional cycles pembrolizumab consolidation every 3 weeks. The primary endpoints are ≥3 pneumonitis rates and remission status. Secondary endpoints include PFS, OS, AEs. The estimated enrolment of 216 patients is planned.

#### H. Lee Moffitt Cancer Center and Research Institute study (NCT03663166)

Open-label multicentre non-randomised phase I/II trial assessing platinum-based cCRT to a dose of 60 Gy with concurrent ipilimumab 1 mg/kg IV on d1 and d22 followed by nivolumab 480 mg IV monotherapy every 4 weeks for up to 1 year. Primary endpoints are unacceptable toxicity status at the end of an 8-week safety observation period and 12-month PFS. An estimated enrolment of 50 participants is planned and an estimated study completion date in 2027.

#### EMD Serono study (NCT03840902)

A multicentre, double-blind randomised controlled study assessing safety and efficacy of platinum-based cCRT with M7824, a novel bifunctional anti-PDL1/TGFβ trap fusion protein followed by M7824. Patients will be randomised to either standard of care platinum-based cCRT to a dose of 60 Gy + placebo followed by durvalumab consolidation or cCRT + M7824 followed by M7824. The primary endpoint is PFS and secondary endpoints including AEs, OS analysis. Enrolment of 350 patients is planned with an estimated completion date set for 2028.

#### CheckMate73L (NCT04026412)

The CheckMate73L is an open-label, multicentre, randomised phase III trial comparing cCRT plus nivolumab followed by nivolumab plus ipilimumab OR cCRT plus nivolumab followed by nivolumab vs. standard of care platinum-based cCRT followed by durvalumab. The co-primary endpoints are PFS and OS. An estimated enrolment of 1400 participants is planned and estimated study completion set for end of 2024.

#### NCI study (NCT04092283)

This study explores the efficacy of cCRT plus durvalumab as concomitant and consolidation therapy or consolidation therapy alone (SoC) in patients with locally advanced, unresectable stage III NSCLC. In this randomised, open--label phase III study, in the concurrent arm participants receive platinum-based cCRT plus durvalumab on d1 & d15 of cycle 1 and d1 of cycle 2 followed by durvalumab every 4 weeks starting within 14 days after the last dose of radiation for 12 cycles. In contrast, in the standard arm, participants receive standard platinum-based cCRT followed by durvalumab also every 4 weeks for a year. The primary endpoint is OS. Secondary endpoints include LRC, PFS, ORR and AEs. Approximately 660 participants will be enrolled with an estimated study completion date set for 10/2028.

## Conclusion

Based on current clinical data, concurrent CRT with maintenance PD-1/PD-L1 inhibition in inoperable stage III NSCLC is a safe and effective multimodal approach with unprecedented median PFS ranging from 16 to 20 months und 2-year OS rates of 60 to 70%. It represents a complex tri-modal approach with high efficacy regarding local and distant tumour control and moderate therapy-related side effects. Hitherto, there is a void with respect to robust predictive biomarkers. Going forward, identification of predictive biological biomarkers for this intensified treatment will be challenging. To optimise development of biomarkers several aspects must be taken in consideration:

A. Reported biomarkers (PD-L1 expression, TMB, IFN gamma gene signature) for immune check-point inhibition as a monotherapy should be tested in the combined multimodal setting.
B. These biomarkers need to be evaluated for correlation with principal patient- and treatment- related factors like gender, age, ECOG, smoking status, tumour volume, histology and type of tumour response (necrotic vs other).
C. It is important to perform a longitudinal analysis of potential biomarkers across all modalities of combined therapy to analyse dynamic and temporary changes before and during every treatment phase.
D. Immunogenic cell death potential of conventional chemotherapeutics (platinum, paclitaxel, pemetrexed etc.), different radiation treatment modes (ultrahypo- vs hypo- vs conventional fractionation) and their combinations need to be determined.
E. It will be necessary to characterise the host immune system in order to evaluate changes during multimodal treatment.
F. Presumably, complexity and plasticity of immune reactions will lead to establishment of a marker panel in order to achieve an effective multimodal treatment personalisation.

Additionally, there has been growing interest in imaging biomarkers particularly with the use of non-invasive molecular imaging agents that can assess expression of immune targets.

In summary, there are a plethora of studies currently assessing CRT plus IO combinations. Incorporation of ICIs with accelerated hypofractionated radiotherapy in treatment concepts could warrant further exploration to cater for patients who present with reduced performance status. Certainly, the question of additional benefit of simultaneous and consolidation ICI with concurrent CRT will be answered in the coming years. However, the duration of ICI consolidation needs to be further explored taking into account cost effectiveness and health economic issues. Finally, it is safe to say that IO is a cornerstone of treatment for inoperable stage III NSCLC and going forward, refinement of this tri-modal concept will only serve to ameliorate patient outcome.

## Data Availability

The datasets used and analysed during the current study are available from the corresponding author on reasonable request.

## Abbreviations

AEs: Adverse events
ATP: Adenosine-5′-triphosphate
AURKA: Aurora kinase A
CI: Confidence interval
CP: Carboplatin/paclitaxel
CRT: Chemoradiotherapy
CTC: Circulating tumour cell
CTLA-4: Cytotoxic T-lymphocyte-associated antigen 4
DAMP: Danger-associated molecular pattern
DC: Dendritic cell
DNA: Deoxyribonucleic acid
ECOG PS: Eastern Cooperative Oncology Group Performance Status
EMA: European Medicines Agency
ETOP: European Thoracic Oncology Platform
FDA: Food and Drug Administration
HMGB1: High mobility group box 1
HR: Hazard ratio
ICB: Immune checkpoint blockade
ICD: Immunogenic cell death
ICI: Immune-checkpoint inhibitor
IFN: Interferon
IgG: Immunoglobulin G
IL: Interleukin
MHC: Major histocompatibility complex
mOS: Median overall survival
NK: Natural killer
NKG2D: Natural killer group 2 member D
NKT: Natural killer T
NSCLC: Non-small cell lung cancer
ORR: Overall response rate
OS: Overall survival
PD: Progressive disease
PD-1: Programmed cell death protein 1
PD-L1: Programmed cell death ligand 1
PFS: Progression-free survival
pre-cCRT: Pre-concurrent chemoradiotherapy
RECIST: Response Evaluation Criteria In Solid Tumours
RNA: Ribonucleic acid
RP: Radiation pneumonitis
RT: Radiotherapy
RTOG: Radiation Therapy Oncology Group
SD: Stable disease
TCR: T cell receptor
TGFβ: Transforming growth factor-β
TMB: Tumour mutational burden
TME: Tumour microenvironment
TMDD: Time to metastatic disease or death
TNF: Tumour necrosis factor
TPX2: Targeting protein for xenopus kinesin-like protein 2
v: Version
yr: Year
